# Supplementary material for: Transporter characterisation reveals aminoethylphosphonate mineralisation as a key step in the marine phosphorus redox cycle
Source: Nat Commun. 2021 Jul 27;12:4554. doi: 10.1038/s41467-021-24646-z (PMC8316502; doi:10.1038/s41467-021-24646-z)
Supplement: Supplementary file 1 — Supplementary Info [file 41467_2021_24646_MOESM1_ESM.pdf]

Transporter characterisation reveals aminoethylphosphonate mineralisation as a key step in the marine phosphorus redox cycle

Andrew R. J. Murphy[1], David J. Scanlan[1], Yin Chen[1], Nathan B. P. Adams[2,3], William A. Cadman[2], Andrew Bottrill[1], Gary Bending[1], John P. Hammond[4], Andrew Hitchcock[2], Elizabeth M. H. Wellington[1], Ian D.E.A. Lidbury[1,5*]

[1]School of Life Sciences, University of Warwick, Gibbet Hill Road, Coventry, UK

[2]Department of Molecular Biology and Biotechnology, University of Sheffield, Sheffield, UK

[3]Nanotemper Technologies GmbH, Flößergasse 4, 81369, Munich, Germany

[4]School of Agriculture, Policy, and Development, University of Reading, Earley Gate, Whiteknights, Reading, UK

[5]Department of Animal and Plant Sciences, University of Sheffield, Sheffield, UK

***Corresponding author:** I.lidbury@sheffield.ac.uk

Table of contents:

Pages 2-4: Supplementary methods

Pages 5-16: Supplementary Figures

Pages 17-22: Supplementary Tables

Supplementary Methods

Generation and complementation of *Pseudomonas* mutants

Regions of genomic DNA at the 5' and 3' end of each deletion target were amplified, along with in some cases the gentamicin resistance cassette from p34S-Gm[1]. Other mutants were constructed to be marker-less, and so lacked this resistance cassette. DNA fragments were ligated into linearised pk18mob*sacB*[2] using the HiFi DNA Assembly Kit (New England Biolabs, Hitchin, UK) according to the manufacturer's instructions. For the construction of plasmids for complementation, genes and their corresponding promoters were cloned into linearised pBBR1McS-km again using the HiFi DNA Assembly Kit.

Plasmids were introduced into *Escherichia coli* S17.1 by electroporation and mobilized into *Pseudomonas* by conjugation. Transconjugants were selected with gentamicin (50 µg ml^{-1}) or kanamycin (50 µg ml^{-1}), and chloramphenicol (10 µg ml^{-1}) was used for counter-selection. Single crossover transconjugants were identified by polymerase chain reaction (PCR), and double crossover mutants selected via plating on LB containing either gentamicin or no antibiotic, and 10% (w/v) sucrose. Homologous recombination was confirmed by PCR and Sanger sequencing. Complemented mutants were selected using kanamycin with chloramphenicol counter-selection.

Enrichment of membrane-associated protein fraction in *P. putida* BIRD-1

40 ml cells were grown to an OD_{600} of 0.8-1.0. A volume equivalent to 5 OD_{600} units was centrifuged at 2000 x *g* for 10 mins, resuspended in 1 ml 50 mM Tris-HCl, pH 7.6, and centrifuged at 2000 x *g* for 10 mins. Pellets were resuspended in 500 µl 200 mM $MgCl_2$, 50 mM Tris-HCl, pH 7.6 and incubated for 30 mins at 30°C with gentle shaking. Cells were cooled on ice for 5 mins and incubated at room temperature for 15 mins, then centrifuged at 8000 x *g* for 10 mins at 4°C. Pellets were washed in 1 ml 50 mM Tris-HCl, pH 7.6, and again centrifuged at 8000 x *g* for 10 mins at 4°C. Pellets were resuspended in 0.5 ml 50 mM Tris-HCl, pH 7.6, and sonicated for 30 s twice, on ice. The solution was centrifuged at 2000 x *g* for 15 mins at 4°C, and supernatants were then ultracentrifuged at 120000 x *g* for 45 mins at 4°C. Following this, the supernatant was discarded and the pellet resuspended in 50 µl LDS buffer (Expedeon) prior to loading 20 µl onto a 4-20% Bis-Tris (2-[Bis(2-hydroxyethyl)amino]-2-(hydroxymethyl)propane-1,3-diol) SDS precast gel (Expedeon).

Detection of Pi efflux

Culture aliquots were centrifuged at 16200 x *g* for 5 mins, and phosphate concentrations in the supernatants were determined according to the method of Chen *et al*.[3] using the modification developed by Christianson and Dunham[4]. Briefly, supernatant was added 1:1 to a 2:1:1:1 solution of dH_2O:6N sulfuric acid:2.5% w/v ammonium molybdate:10% w/v ascorbic acid, incubated at 37 °C for 90 mins, and absorbance was read at 820 nm.

Production, purification and characterisation of recombinant *S. stellulata* AepX

The *S. stellulata* DSM 5886 *aepX* gene lacking the sequence encoding the predicted N-terminal signal peptide (28 aa) and stop codon was amplified from *S. stellulata* DSM 5886 genomic DNA using primer pair pET21:AepXSs_Fwd and pET21:AepXSs_Rev (Supplementary table 4). The resulting product was inserted between the NdeI and XhoI sites of pET21a(+) (Novagen) using the HiFi DNA Assembly Kit (New England Biolabs, Hitchin, UK) according to the manufacturer's instructions. Plasmid

pET21a::*aepX*^{Ss} was sequence verified by automated DNA sequencing (Eurofins, Germany) and introduced to *E. coli* BL21(DE3) for over-production of recombinant AepX with a C-terminal hexahistidine tag.

E. coli BL21(DE3) harbouring pET21a::*aepX*^{Ss} was grown in LB broth with 100 µg mL^{-1} ampicillin in a culture volume of 1 L contained within a 2 L baffled flask. Following inoculation from a 5 mL overnight starter culture, cells were grown at 37 °C and 250 rpm shaking until they reached an OD_{600} of ~0.6, at which point isopropyl β-D-1-thiogalactopyranoside was added to final concentration of 0.4 mM and the culture was incubated shaking (250 rpm) overnight at 18 °C. Cells were harvested by centrifugation at 5000 x *g* for 15 mins at 4 °C and resuspended in ~50 mL of 25 mM HEPES (4-(2-hydroxyethyl)-1-piperazineethanesulfonic acid) buffer pH 7.5 containing 500 mM NaCl and 5 mM imidazole (binding buffer). A small spatula of DNase I was added to the suspension and cells were broken by sonication on ice (6 × 30 s bursts with 30 s intervals between) prior to removal of insoluble debris by centrifugation at 56000 x *g* for 20 min at 4 ˚C. Proteins were purified by immobilised Ni-affinity chromatography on a 5 ml Chelating SepharoseTM Fast Flow resin column (GE Healthcare, UK). Bound protein was washed with binding buffer containing 20 mM imidazole and then eluted with 25 mM HEPES pH 7.5 containing 100 mM NaCl and 400 mM imidazole. Eluted protein was concentrated to a volume of 2 mL in a Vivaspin centrifugal concentrator and AepX was further purified on a 22 mL Superdex S200 Increase gel filtration column (GE Healthcare) in 20 mM HEPES pH 7.5 containing 200 mM NaCl. AepX eluted as a single peak and was adjudged pure by SDS-PAGE analysis. Pooled peak fractions were stored at 4 ˚C.

The binding affinity of AepX for phosphonate ligands was determined using Microscale Thermophoresis (MST) with a Monolith NT.115 instrument (NanoTemper Technologies, Germany). AepX (100 nM) was labelled with 2nd Generation RED-tris-NTA dye (Nanotemper Technologies, Germany) in 20 mM HEPES pH 7.5 with 200 mM NaCl and 0.005% (v/v) Tween-20 (MST buffer) according to the manufacturer's instructions. Ligand stocks (purchased from Sigma) were prepared at appropriate concentrations in MST buffer. A 10 µl, 16 point, 2-fold ligand dilution series was prepared, and subsequently mixed with 10 µl of labelled protein. 4 µL of each protein-ligand mixture was loaded into Monolith NT.115 Premium Capillaries (Nanotemper Technologies, Germany) and thermophoresis was measured at 22 ˚C for 22 s with 60 % LED power and High MST power. Data from at least two independent titrations were combined and analysed using the MO.Affinity Analysis software version 2.3 (Nanotemper Technologies, Germany), using the early thermophoresis data at 1 – 2.5 seconds in the experiment for analysis. Isotherms were fitted to a single binding site model (Equation 1) where [l] is concentration of ligand and data plotted using Igor Pro version 8.04 (Wavemetrics Inc., USA).

$$f(\mathrm{l}) = \mathrm{Unbound} + \frac{(\mathrm{Bound}-\mathrm{Unbound})\times([\mathrm{l}]+[\mathrm{protein}]+K_d-\sqrt{([\mathrm{l}]+[\mathrm{protein}]+K_d)^2-4\times[\mathrm{l}]\times[\mathrm{protein}]}}{2\times[\mathrm{protein}]}$$

Equation 1. Single binding site model used to determine dissociation constants.

References

1. Dennis, J.J. and G.J. Zylstra, *Plasposons: Modular self-cloning minitransposon derivatives for rapid genetic analysis of Gram-negative bacterial genomes.* Applied and Environmental Microbiology, 1998. **64**(7): p. 2710-2715.
2. Schäfer, A., et al., *Small mobilizable multi-purpose cloning vectors derived from the Escherichia coli plasmids pK18 and pK19: selection of defined deletions in the chromosome of Corynebacterium glutamicum.* Gene, 1994. **145**(1): p. 69-73.
3. Chen, P.S., T.Y. Toribara, and H. Warner, *Microdetermination of Phosphorus.* Analytical Chemistry, 1956. **28**(11): p. 1756-1758.
4. Christianson, C. and M. Dunham. *Phosphate Assay*. 2005; Available from: https://dunham.gs.washington.edu/MDphosphateassay.htm.

Supplementary Figures

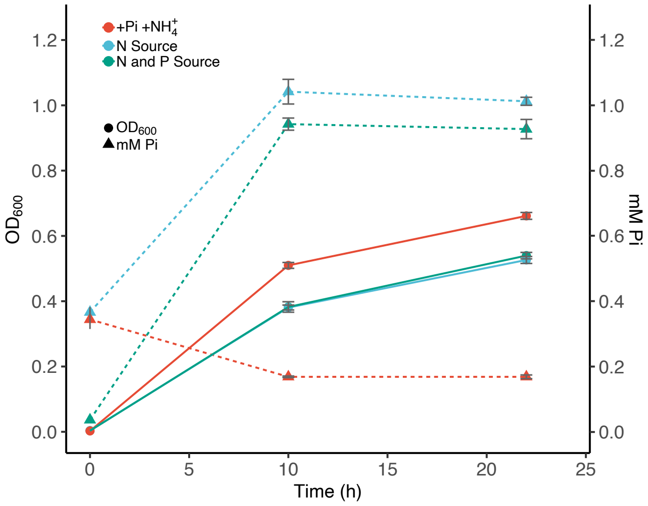

Figure S1. Growth of *Pseudomonas putida* BIRD-1 using 2AEP as the sole N or sole N and sole P source. Pi efflux (triangles and dashed lines) and growth (circles and solid lines) on 2AEP as the sole N (blue) or sole N and sole P source in *P. putida* BIRD-1 (green). A positive control was also established where orthophosphate (Pi) and ammonium (NH_4^+) were the sole P and N sources respectively (red). All conditions were performed in triplicate; error bars represent the standard deviation of the mean.

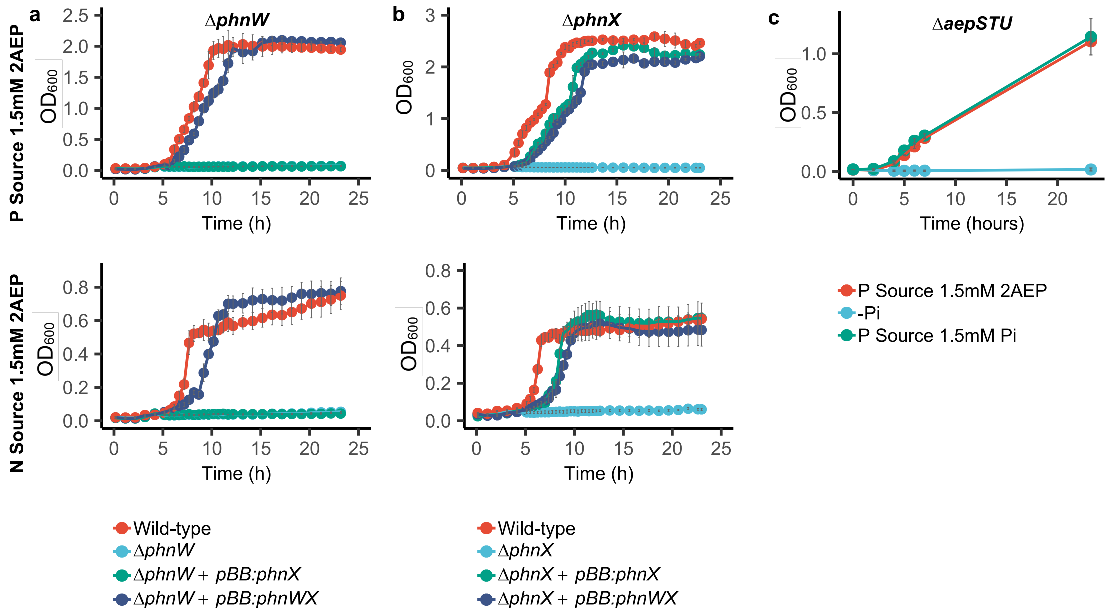

Figure S2 Growth curves of WT and mutant strains of *Pseudomonas putida* BIRD-1 on 2AEP as sole N or P source. (**a**) Shows WT, Δ*phnW* and Δ*phnW in trans* complemented with *phnW(X)* grown with 1.5 mM 2AEP as the sole P source (upper panel) or N source (lower panel). (**b**) Shows WT, Δ*phnX* and Δ*phnX in trans* complemented with *phnWX* grown with 1.5 mM 2AEP as the sole P source (upper panel) or N source (lower panel). (**c**) Shows Δ*aepSTU* grown in the absence of Pi or with 1.5 mM 2AEP or Pi as sole P source. Error bars show standard deviations of the mean from four biological replicates.

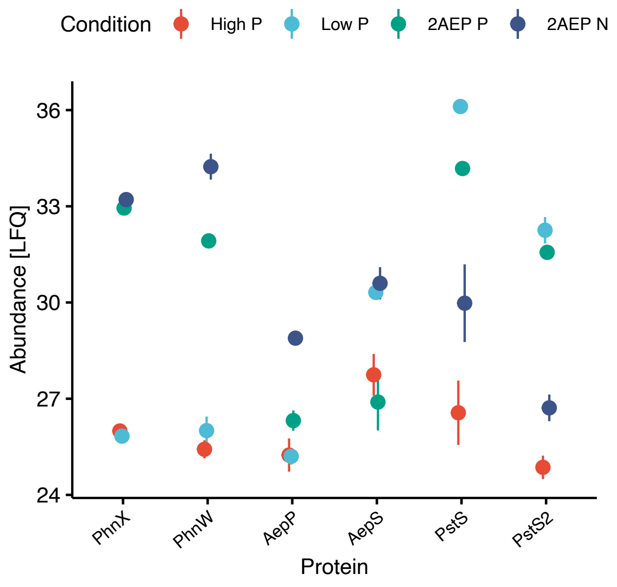

Figure S3. Assessment of *Pseudomonas putida* BIRD-1 membrane-associate protein fraction. Quantification (LFQ_{Log2} values) of PhnWX, candidate 2AEP transport proteins and phosphate binding proteins (PstS) in Δ*aepXVW*::*gm* in response to growth on 2AEP (n=3) as sole P or N source. High and low P controls used orthophosphate (Pi) as the sole P source. Error bars show standard deviation of the mean.

```
GlpT            MLSIFKPAPHKARLPAAEIDPTYRRLRWQIFLGIFFGYAAYYLVRKNFALAMPYLVEQ-G
AepP            ---------MNQSLAA------FKRWRIQIFAITWLAYAAFYFTRKAFSVAKLGIAEDPG
                          :  *.*      :.* * ***   ::.***:*:.** *::*   :.*: *

GlpT            FSRGDLGFA-LSGISI-AYGFSKFIMGSVSDRSNPRVFLPAGLILAAAVMLFMGFVPWAT
AepP            FMLDKAAMANLDAIYLAAYAVGQFTWGMLADRFGPRVVVLGGLLISAAAAVVMG----SY
                *  .. .:* *..* : **...:*  * ::** .***.: .**:::**. :.**    :

GlpT            SSIAVMFVLLFLCGWFQGMGWPPCGRTMVHWWSQKERGGIVSVWNCAHNVGGGI-PPLLF
AepP            ATFPIFATCMLVQGLAQSTGWAGLCKNIGSFFPASQRGRVLGLWSSCYAFGGLVASPFAG
                :::.:: . ::: *  *. **.   ..:  ::. .:** ::.:*...: .** : .*:

GlpT            LLGMAWFNDWHAALYMPAFCAILVALFAFAMMRDTPQSCGLPPIEEYKNDYPDDYNEKAE
AepP            WWAYTLVGNWHAAFFSSAAVVALVAVLFFFLQRNKPEDVGLPAVE------PEPQSMAPA
                  . : ..:****:: .*  . ***:: * : *:.*:. ***.:*      *:  .  .

GlpT            QELTAKQIFMQYVLPNKLLWYIAIANVFVYLLRYGILDWSPTY-LKEVKHFALDKSSWAY
AepP            GSLCSVWAPLREILRNRTVLTLGLAYFLLKPARYAILLWGPVIVFEQMPSVGKVGAAIIP
                 .* : .  :. :* *. :  :.:* .::   **.** *.*.  ::::  ..   ::

GlpT            FLYEYAGIPGTLLCGWMSDKVFRGNRGATGVFFMTLVTIATIVYWMNP--AGNPTVDMIC
AepP            TAFELAGLLGPIMIGLASDKLFGARRMPACVISLVLLTV-TLALFMAAMHTGSVLLVVVL
                  :* **: *.:: *  ***:* ..* .: *: :.*:*: *:. :* .  :*.  : ::

GlpT            MIVIGFLIYGP-VMLIGLHALELAPKKAAGTAAGFTGLFGYLGGSVAASAIVGYTVDFFG
AepP            LFVMGLTLYGPDSMISGAAAIDFGTAKAGATAAGFVNGCGSVGAVLG-----GLLPGYFD
                ::*:*: :***  *: *  *:::.. **..*****..  * :*. :.     *   .:*.

GlpT            WDGGFMVMIGGSILAVILLIVVMIGEKRRHEQLLQERNGG---------
AepP            GVTVFIVFAGCALFSALVLLPHWNSRPASSAQGTDVAPNTSMAIKPLRT
                    *:*: * ::::.::*:    .      *  :   .
```

Figure S4. ClustalOmega alignment of GlpT, the glycerol-3-phosphate/phosphate (G3P/Pi) antiporter, and AepP protein sequences. Residues that have been shown to bind with the phosphate moiety of G3P, and/or Pi are shown in red if identical, in orange if related, and in blue if unrelated. Residues involved in binding the glycerol moiety of G3P are shown in green.

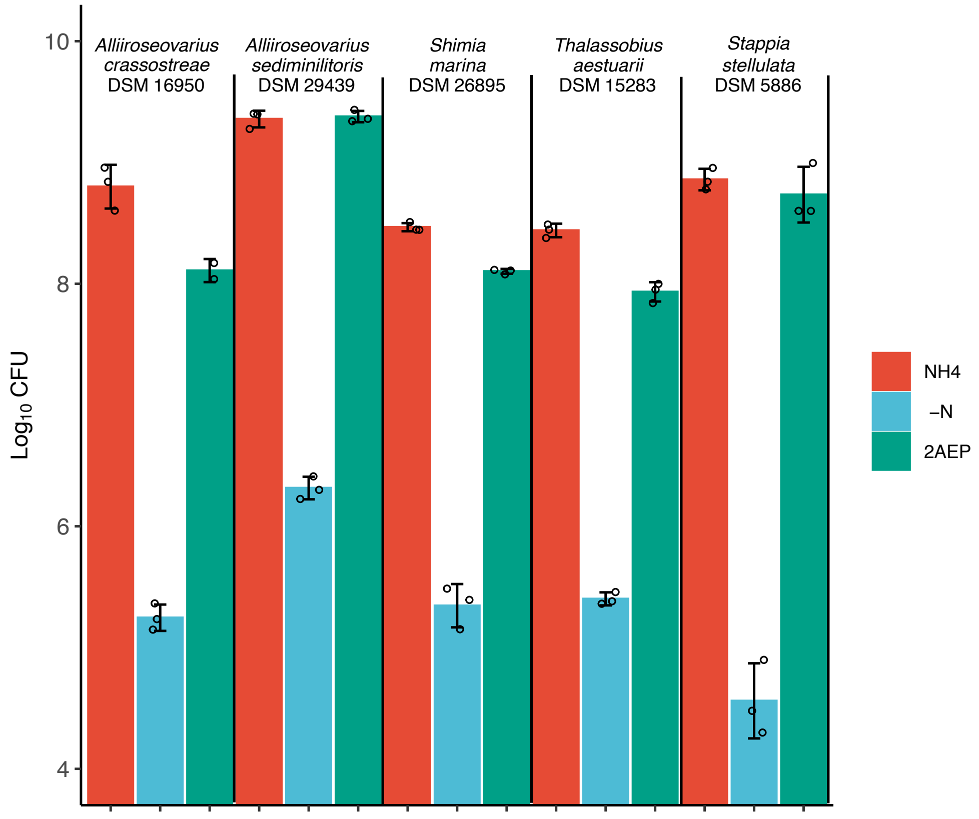

Figure S5. Enumeration of *Roseobacter* strains grown on 2AEP as a sole nitrogen source. Colony forming units were obtained for various *Roseobacter* strains grown on either 1.5 mM NH_4 or 1.5 mM 2AEP in addition to a negative control containing no exogenous nitrogen source. Cultures were sampled between 36-60 hours growth. Results presented are the mean of triplicate cultures. Error bars denote standard deviation.

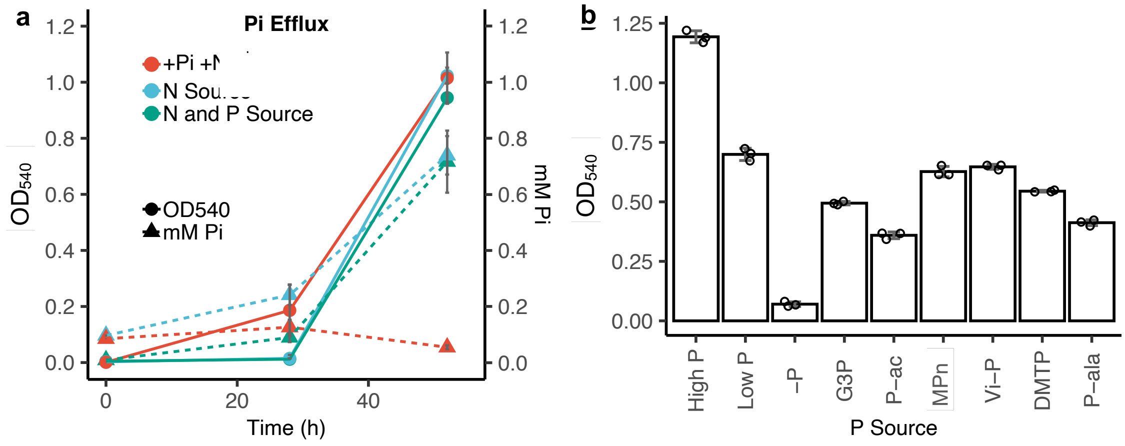

Figure S6. Growth of *Stappia stellulata* DSM5886 on various phosphonate compounds. (**a**) Pi efflux (triangles and dashed lines) and growth (circles and solid lines) on 2AEP as the sole N source (blue), or sole N and P source (green). A positive control was also established where orthophosphate (Pi) and ammonium (NH_4^+) were the sole P and N sources respectively (red). All conditions were performed in triplicate; error bars represent the standard deviation of the mean. (**b**) Growth on various P sources. High P = 1 mM Pi, Low P = 0.1 mM Pi, -P = No P Control, G3P = Glycerol-3-phosphate, P-ac = Phosphonoacetate, MPn = Methylphosphonate, Vi-P = Vinylphosphonate, DMTP = Dimethylthiophosphonate, P-ala = Phosphonoalanine. All organic P sources are 0.1 mM. Values are the mean of three replicates; error bars are the standard deviation of the mean.

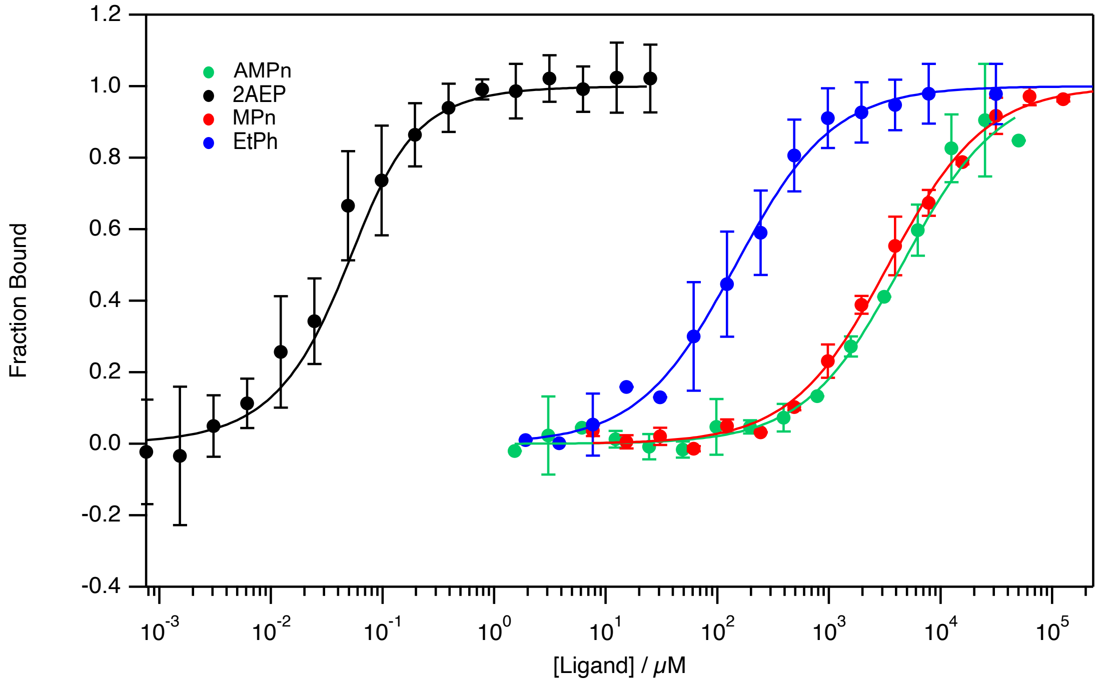

Figure S7. Binding affinities for selected phosphonates by the *Stappia stellulata* AepX substrate binding protein determined by microscale thermophoresis. Results presented are the mean of n=3 (2AEP) or n=2 (MPn, methylphosphonate; EtPh, ethylphosphonate; AMP, aminomethylphosohonate) titrations and error bars denote standard deviation. The fraction of ligand bound to protein was calculated using the MO.affinity analysis software (Nanotemper Technologies, Garmany) by converting Fnorm values.

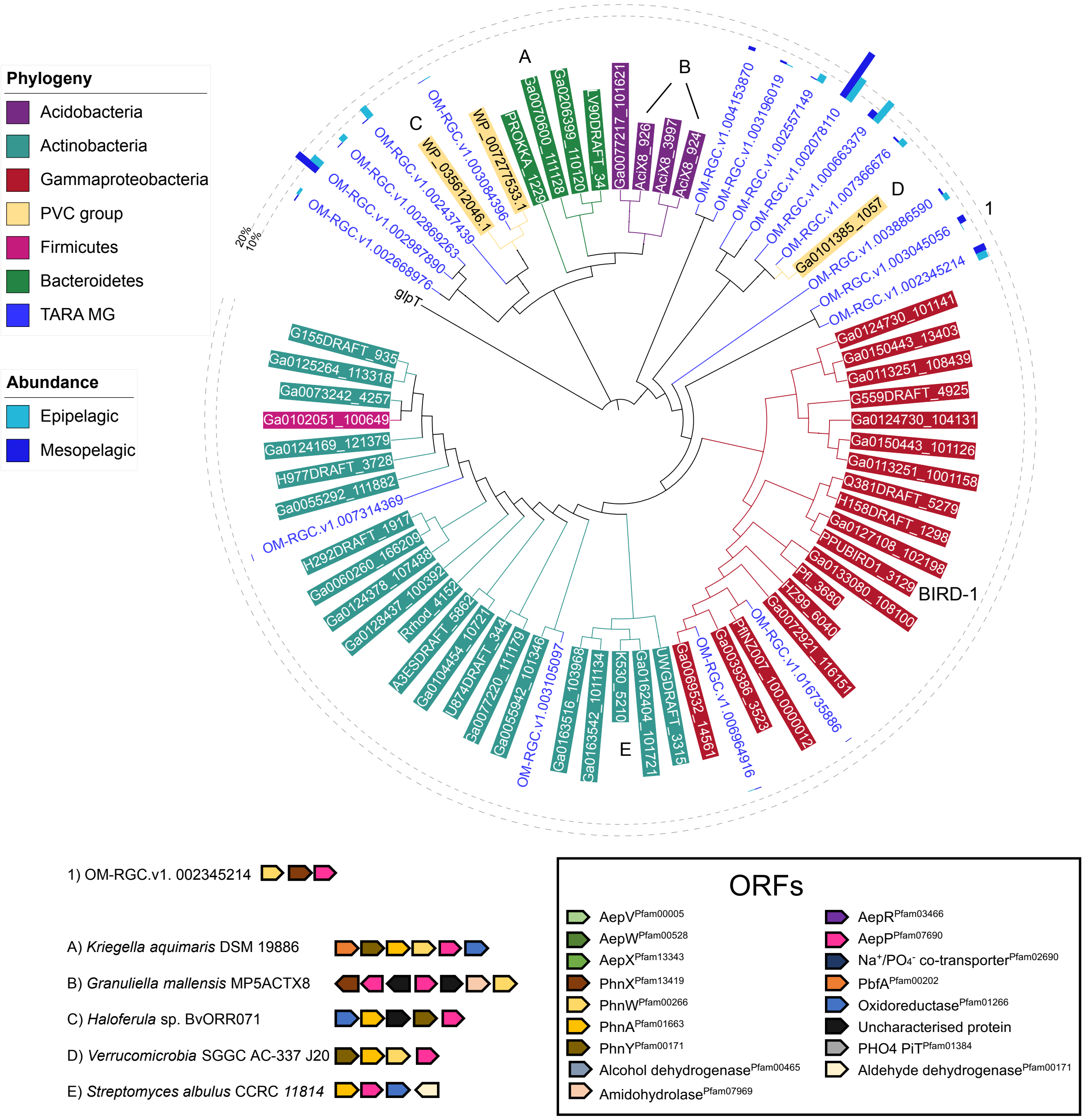

Figure S8. Phylogenetic and genomic analyses of AepP in marine and terrestrial bacteria. The genetic neighbourhood for selected *aepP* homologs is presented adjacent to the tree. Numbers indicate environmental OTUs and letters indicate isolates or MAGs/SAGs. Tree topology and branch lengths were calculated by maximum likelihood using the LG+F+I+G4 model of evolution for amino acid sequences based on 301 sites in IQ-TREE software. A consensus tree was generated using 1000 bootstraps. Branches representing isolates or MAGs/SAGs are colour-coded based on their phylogenetic affiliation (see legends). Branches and identifiers for representative environmental OTU sequences (clustered at 0.8) retrieved from the TARA Oceans database (blue) are also highlighted. The outer ring denotes the relative abundance of environmental OTUs using the same colour scheme; relative abundance values of 10% (dashed line) and 20% (filled line) are shown for scale; *P. putida* BIRD-1 AepP is labelled.

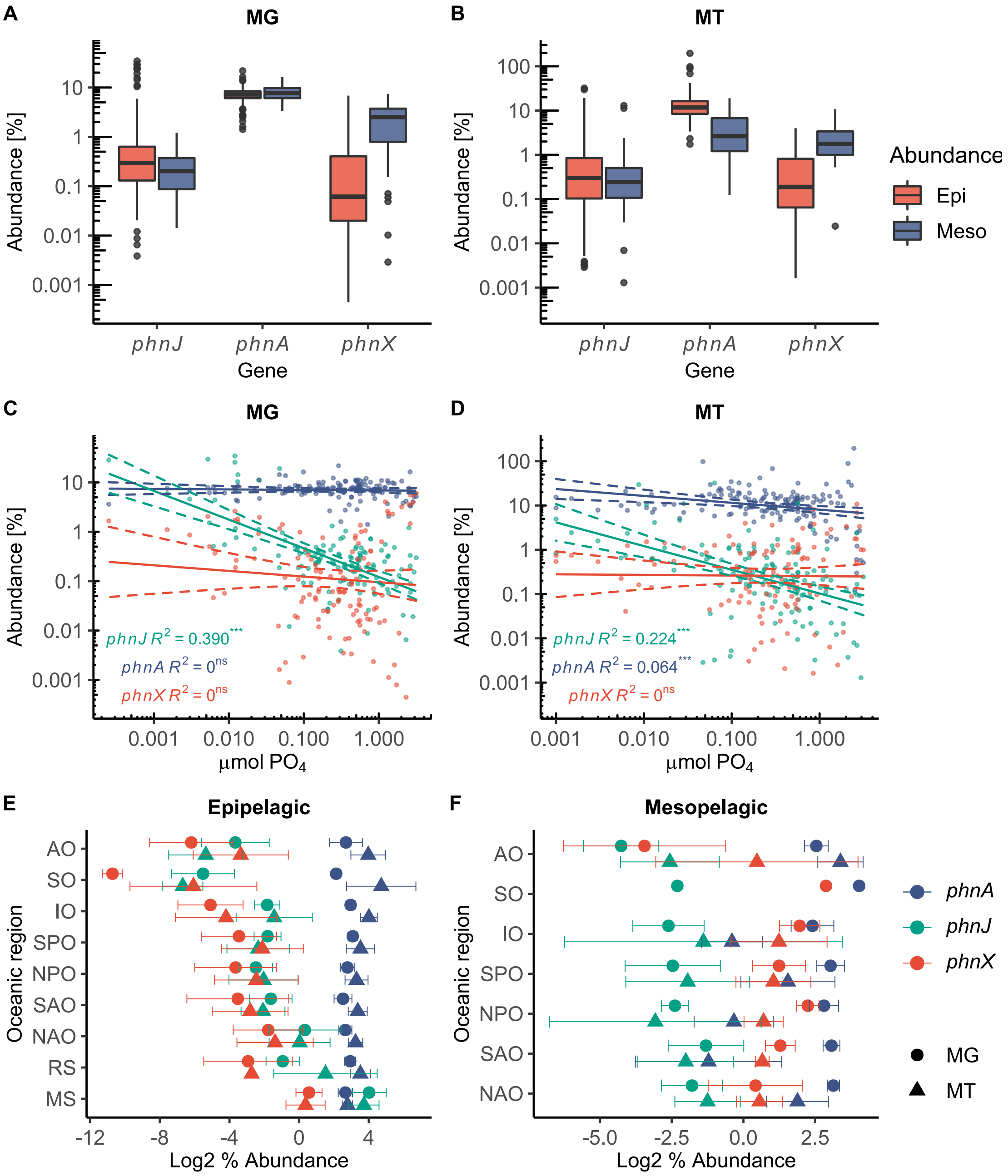

Figure S9. Metagenomic (A) and metatranscriptomic (B) abundance of marker genes for 2AEP degradation within the TARA ocean dataset. Data is split between epipelagic (Epi – Red) and mesopelagic (Meso – Blue) waters. Abundance is normalised as a percentage of the median abundance of 10 single copy marker genes within either OM-RGCv2+G (MG) or OM-RGCv2+T (MT). MG (**A**) epipelagic: *phnJ n* = 137, *phnA n* = 137, *phnX n* = 126, mesopelagic: *phnJ n* = 43, *phnA n* = 43, *phnX n* = 43, where *n* equals the number of biologically independent sampling sites where the genes were located. MT (**B**) epipelagic: *phnJ n* = 133, *phnA n* = 154, *phnX n* = 104, mesopelagic: *phnJ n* = 31, *phnA n* = 33, *phnX n* = 30, where *n* equals the number of biologically independent sampling sites where the transcripts were located. The relationship between the standing stock Pi concentration and degradation marker abundance, analysed by linear regression of Log_{10} Pi concentration and Log_{10} gene/transcript abundance, in the MG (**C**) (*phnJ* R^2 = 0.390, p = 7.73e^{-16}, *phnA* R^2 = -0.006, p = 0.636, *phnX* R^2 = 0.000, p = 0.333), and MT (**D**) (*phnJ* R^2 = 0.224, p = 3.55e^{-9}, *phnA* R^2 = 0.064, p = 8.15e^{-4}, *phnX* R^2 = -0.009, p = 0.893) R^2 values are shown. *** = p<0.001, ns =non-significant. Log2 abundance of

phosphonate degradation marker genes within metagenome (circles) and metatranscriptome (triangles) in Epipelagic (**E**) and Mesopelagic (**F**) waters within various oceanic regions. Circles/triangles denote mean Log_2 abundance values, error bars denote standard deviation of the mean. *phnA* (blue), *phnJ* (green), *phnX* (red). AO = Arctic Ocean, SO = Southern Ocean, IO = Indian Ocean, SPO = South Pacific Ocean, NPO = North Pacific Ocean, SAO = South Atlantic Ocean, NAO = North Atlantic Ocean, RS = Red Sea, MS = Mediterranean Sea. Epipelagic (**E**) *phnJ* AO MG *n* = 29, MT *n* = 14, SO MG *n* = 3, MT *n* = 4, IO MG *n* = 21, MT *n* = 19, SPO MG *n* = 25, MT *n* = 34, NPO MG *n* = 11, MT *n* = 20, SAO MG *n* = 14, MT *n* = 15, NAO MG *n* = 16, MT *n* = 17, RS MG *n* = 6, MT *n* = 3, MS MG *n* = 12, MT *n* = 7, *phnA* AO MG *n* = 29, MT *n* = 28, SO MG *n* = 3, MT *n* = 8, IO MG *n* = 21, MT *n* = 19, SPO MG *n* = 25, MT *n* = 35, NPO MG *n* = 11, MT *n* = 20, SAO MG *n* = 14, MT *n* = 17, NAO MG *n* = 16, MT *n* = 17, RS MG *n* = 6, MT *n* = 3, MS MG *n* = 12, MT *n* = 7, *phnX* AO MG *n* = 21, MT *n* = 11, SO MG *n* = 2, MT *n* = 2, IO MG *n* = 20, MT *n* = 9, SPO MG *n* = 25, MT *n* = 26, NPO MG *n* = 11, MT *n* = 19, SAO MG *n* = 13, MT *n* = 13, NAO MG *n* = 16, MT *n* = 16, RS MG *n* = 6, MT *n* = 2, MS MG *n* = 12, MT *n* = 6. Mesopelagic (**F**) *phnJ* AO MG *n* = 9, MT *n* = 7, SO MG *n* = 1, MT *n* = 0, IO MG *n* = 6, MT *n* = 4, SPO MG *n* = 9, MT *n* = 9, NPO MG *n* = 5, MT *n* = 5, SAO MG *n* = 5, MT *n* = 2, NAO MG *n* = 8, MT *n* = 5, *phnA* AO MG *n* = 9, MT *n* = 7, SO MG *n* = 1, MT *n* = 0, IO MG *n* = 6, MT *n* = 4, SPO MG *n* = 9, MT *n* = 9, NPO MG *n* = 5, MT *n* = 5, SAO MG *n* = 5, MT *n* = 2, NAO MG *n* = 8, MT *n* = 6, MT *n* = 7, *phnX* AO MG *n* = 9, MT *n* = 7, SO MG *n* = 1, MT *n* = 0, IO MG *n* = 6, MT *n* = 4, SPO MG *n* = 9, MT *n* = 9, NPO MG *n* = 5, MT *n* = 5, SAO MG *n* = 5, MT *n* = 2, NAO MG *n* = 8, MT *n* = 5. In **a**-**b** data are represented as boxplots, where the middle line is the median and the upper and lower hinges correspond to the first and third quartiles. The upper whisker extends from the upper hinge to the largest value that is no more than 1.5×IQR (inter-quartile range) from the upper hinge, and the lower whisker extends from the lower hinge to the smallest value that is no further than 1.5×IQR from the lower hinge. Data beyond the ends of the whiskers are outlying points that are plotted individually.

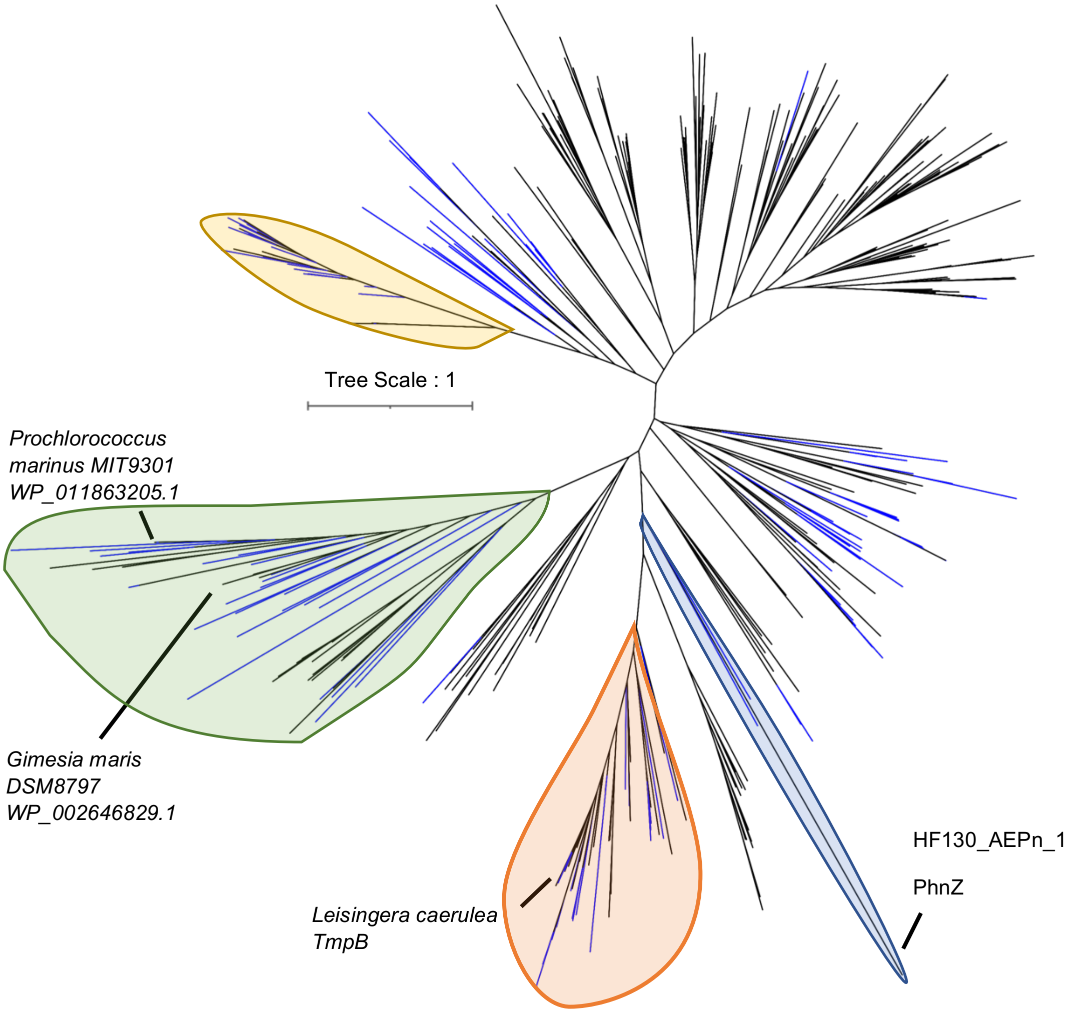

Figure S10. Phylogenetic tree of PhnZ homologs. Environmental sequences are shown in blue, Refseq sequences are shown in black. Clades of interest are highlighted based on examination of the gene neighbourhoods of the Refseq PhnZ homologs contained within. The (hydroxy-)methylphosphonate specific PhnZ clade is highlighted in green, the (N)-trimethyl-2-aminophosphonate specific TmpB clade is highlighted in orange, and the 2AEP specific clade is highlighted in blue. Many of the remaining abundant sequences fall within the clade highlighted in gold, but the genetic context of PhnZ sequences from Refseq organisms within is not conserved, and no function has been demonstrated.

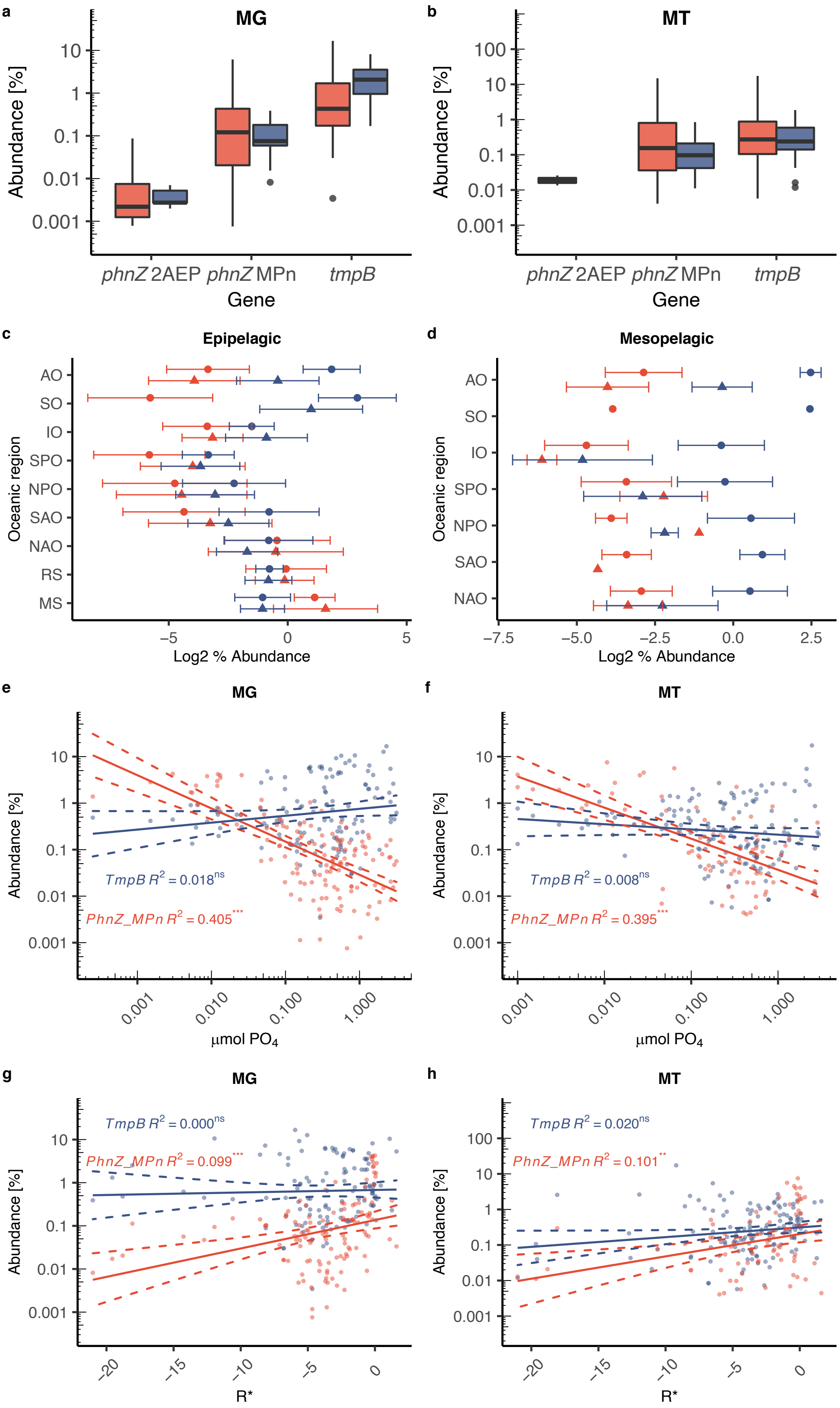

a
MG
Abundance [%]
phnZ 2AEP
phnZ MPn
tmpB
Gene
b
MT
c
Epipelagic
Oceanic region
AO
SO
IO
SPO
NPO
SAO
NAO
RS
MS
Log2 % Abundance
d
Mesopelagic
e
MG
TmpB $R^2 = 0.018^{ns}$
PhnZ_MPn $R^2 = 0.405^{***}$
μmol PO4
f
MT
TmpB $R^2 = 0.008^{ns}$
PhnZ_MPn $R^2 = 0.395^{***}$
g
MG
TmpB $R^2 = 0.000^{ns}$
PhnZ_MPn $R^2 = 0.099^{***}$
R*
h
MT
TmpB $R^2 = 0.020^{ns}$
PhnZ_MPn $R^2 = 0.101^{**}$

Figure S11. PhnZ abundance in the TARA oceans dataset. Metagenomic (**a**) and metatranscriptomic (**b**) abundance of three *phnZ* clades (*phnZ* 2AEP = 2AEP specific, *phnZ* MPn = methlyphosphonate specific, *tmpB* = (*N*)-trimethyl-2-aminoethylphosphonate specific) within the TARA ocean dataset. Data is split between epipelagic (red), and mesopelagic (blue) waters. Abundance is normalised as a percentage of the median abundance of 10 single copy marker genes within either OM-RGCv2+G (MG) or OM-RGCv2+T (MT). MG (**a**) epipelagic: *phnZ* 2AEP *n* = 4, *phnZ* MPn *n* = 134, *tmpB n* = 137, mesopelagic: *phnZ* 2AEP *n* = 5, *phnZ* MPn *n* = 43, *tmpB n* = 43, where *n* equals the number of biologically independent sampling sites where the genes were located. MT (**b**) epipelagic: *phnZ* 2AEP *n* = 2, *phnZ* MPn *n* = 82, *tmpB n* = 148, mesopelagic: *phnZ* 2AEP *n* = 2, *phnZ* MPn *n* = 19, *tmpB n* = 24, where *n* equals the number of biologically independent sampling sites where the transcripts were located. Abundance (Log2 % abundance relative to median abundance of 10 single copy core genes) of *phnD*, *aepX*, *aepP* in MG (circles) and MT (triangles) in epipelagic (**c**) and mesopelagic (**d**) waters, split by oceanic region. *phnZ*_MPn (red), *tmpB* (blue). AO = Arctic Ocean, SO = Southern Ocean, IO = Indian Ocean, SPO = South Pacific Ocean, NPO = North Pacific Ocean, SAO = South Atlantic Ocean, NAO = North Atlantic Ocean, RS = Red Sea, MS = Mediterranean Sea. Circles/triangles are mean values of Log_2 abundance, error bars represent standard deviation of the mean. Epipelagic (**c**) *phnZ* MPn AO MG *n* = 27, MT *n* = 26, SO MG *n* = 3, MT *n* = 8, IO MG *n* = 21, MT *n* = 18, SPO MG *n* = 24, MT *n* = 35, NPO MG *n* = 11, MT *n* = 20, SAO MG *n* = 14, MT *n* = 17, NAO MG *n* = 16, MT *n* = 17, RS MG *n* = 6, MT *n* = 3, MS MG *n* = 12, MT *n* = 7, *tmpB* AO MG *n* = 29, MT *n* = 28, SO MG *n* = 3, MT *n* = 8, IO MG *n* = 21, MT *n* = 19, SPO MG *n* = 25, MT *n* = 35, NPO MG *n* = 11, MT *n* = 20, SAO MG *n* = 14, MT *n* = 17, NAO MG *n* = 16, MT *n* = 17, RS MG *n* = 6, MT *n* = 3, MS MG *n* = 12, MT *n* = 7. Mesopelagic (**d**) *phnZ* MPn AO MG *n* = 9, MT *n* = 7, SO MG *n* = 1, MT *n* = 0, IO MG *n* = 6, MT *n* = 4, SPO MG *n* = 9, MT *n* = 9, NPO MG *n* = 5, MT *n* = 5, SAO MG *n* = 5, MT *n* = 2, NAO MG *n* = 8, MT *n* = 6, MT *n* = 7, *tmpB* AO MG *n* = 9, MT *n* = 7, SO MG *n* = 1, MT *n* = 0, IO MG *n* = 6, MT *n* = 4, SPO MG *n* = 9, MT *n* = 9, NPO MG *n* = 5, MT *n* = 5, SAO MG *n* = 5, MT *n* = 2, NAO MG *n* = 8, MT *n* = 6, MT *n* = 7. The relationship between the standing stock Pi concentration and *phnZ* abundance, analysed by linear regression of Log_{10} Pi concentration and Log_{10} gene/transcript abundance, in the MG (**e**) (*phnZ* MPn R^2 = 0.405, p = 2.354e^{-16}, *tmpB* R^2 = 0.018, p = 0.066), and MT (**f**) (*phnZ* MPn R^2 = 0.395, p = 2.707e^{-10}, *tmpB* R^2 = 0.008, p = 0.1426) R^2 values are shown. *** = p<0.001, ns =non-significant. The relationship between R*, a measure of N vs P limitation defined as the sum of standing stock nitrate plus nitrite concentration minus 16x standing stock Pi concentration, and transporter abundance, analysed by linear regression of R* and Log_{10} gene/transcript abundance, in the MG (**g**) (*phnZ* MPn R^2 = 0.099, p = 2.8e^{-4}, *tmpB* R^2 = -0.007, p = 0.7158) and MT (**h**) (*phnZ* MPn R^2 = 0.101, p = 3.057e^{-3}, *tmpB* R^2 = 0.021, p = 0.051). *phnZ*_MPn (red), *tmpB* (blue), ns = not significant, ** = p < 0.01, *** = p < 0.001. 95% confidence intervals are shown by dashed lines. In **a**-**b** data are represented as boxplots, where the middle line is the median and the upper and lower hinges correspond to the first and third quartiles. The upper whisker extends from the upper hinge to the largest value that is no more than 1.5×IQR (inter-quartile range) from the upper hinge, and the lower whisker extends from the lower hinge to the smallest value that is no further than 1.5×IQR from the lower hinge. Data beyond the ends of the whiskers are outlying points that are plotted individually.

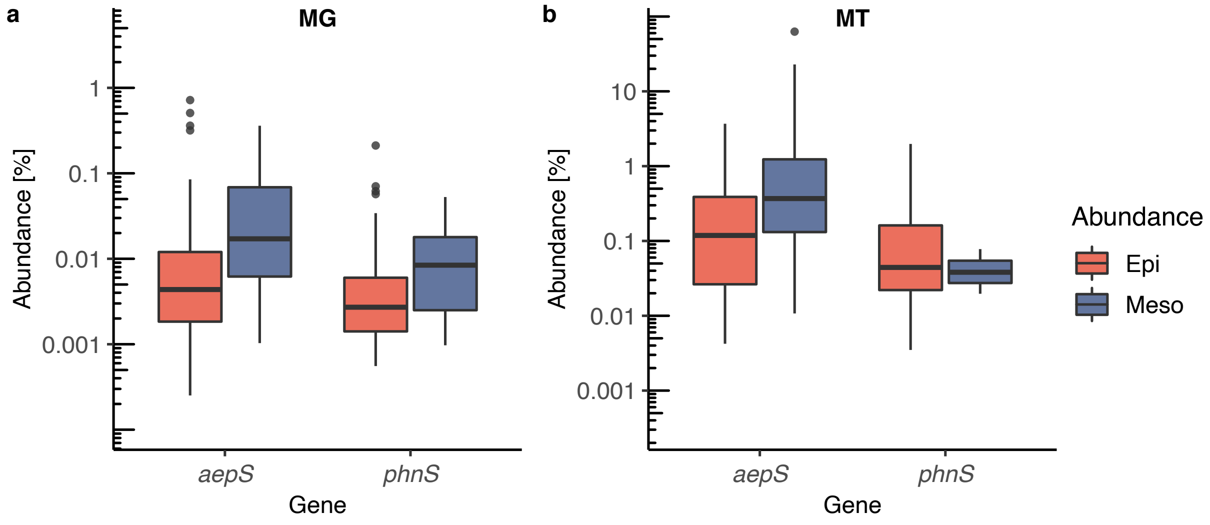

Figure S12. Metagenomic (a) and metatranscriptomic (b) abundance of the alternate 2AEP ABC transporter substrate binding proteins AepS and PhnS within the TARA oceans dataset. Data is split between epipelagic (Epi – Red), and mesopelagic (Meso – Blue) waters. Abundance is normalised as a percentage of the median abundance of 10 single copy marker genes within either OM-RGCv2+G (MG) or OM-RGCv2+T (MT). MG (**a**) epipelagic: *aepS n* = 88, *phnS n* = 48, mesopelagic: *aepS n* = 35, *phnS n* = 29, where *n* equals the number of biologically independent sampling sites where the genes were located. MT (**b**) epipelagic: *aepS n* = 91, *phnS n* = 25, mesopelagic: *aepS n* = 26, *phnS n* = 3, where *n* equals the number of biologically independent sampling sites where the transcripts were located In **a**-**b** data are represented as boxplots, where the middle line is the median and the upper and lower hinges correspond to the first and third quartiles. The upper whisker extends from the upper hinge to the largest value that is no more than 1.5×IQR (inter-quartile range) from the upper hinge, and the lower whisker extends from the lower hinge to the smallest value that is no further than 1.5×IQR from the lower hinge. Data beyond the ends of the whiskers are outlying points that are plotted individually.

Supplementary Tables

Table S1 Final growth yields (mean OD_{540} ± standard deviation) of *Stappia stellulata* (DSM 5886), *Alliiroseovarius crassostreae* (DSM 16950), *Alliiroseovarius sediminilitoris* (DSM 29439), *Thalassobius aestuarii* (DSM 15283), and *Shimia marina* (DSM26895) on 2AEP as sole N or P source with corresponding negative and positive controls. All conditions were performed in triplicate.

Organism	+Pi	$+NH_4$	-Pi	$-NH_4$	2AEP (P Source)	2AEP (N Source)
DSM 16950	0.90±0.018	0.776±0.044	0.082±0.012	0.018±0.011	1.050±0.018	0.535±0.039
DSM 29439	1.100±0.037	0.257±0.004	0.108±0.003	0.026±0.007	0.748±0.019	0.239±0.008
DSM 26895	1.045±0.020	0.397±0.035	0.157±0.019	0.045±0.008	0.827±0.013	0.347±0.004
DSM 15283	1.003±0.030	0.549±0.041	0.179±0.052	0.059±0.000	0.869±0.094	0.487±0.020
DSM 5886	1.01±0.07	0.680±0.016	0.07±0.008	0.026±0.004	0.980±0.005	0.816±0.080

Table S2 Phosphonate transport and catabolism gene complement of strains used in this paper. Strains used for growth curves and/or proteomics are highlighted in bold. + = present, - = absent.

Organism	PhnWAY	PhnWX	PhnJ	PhnY*Z	AepVWX	PhnCDE	PhnSTUV	AepP
***Alliiroseovarius crassostreae* DSM 16950**	+	-	-	-	+	-	-	-
***Aliiroseovarius sediminilitoris* DSM 29439**	+	-	-	-	+	-	-	-
***Shimia marina* DSM 26895**	+	-	+	-	+	+	-	-
***Thalassobius aestuarii* DSM 15283**	+	-	+	-	+	+	-	-
***Stappia stellulata* DSM 5886**	+	-	+	-	+	+	-	-
***Pseudomonas putida* BIRD-1**	-	+	-	-	+	-	-	+
***Pseudomonas flourescens* SBW25**	-	+	+	-	+	+	-	-
Chitinibacter tainanensis DSM 15459	-	+	-	-	+	-	-	-
Paraburkholderia insulsa LMG 28183	+	-	+	-	+	+	+	-
Alicycliphilus denitrificans BC	+	-	-	-	+	-	-	-
Oceanimonas smirnovii ATCC BAA-899	-	+	-	-	+	-	-	-
Burkholderia cepacia RB-39	+	-	-	-	+	-	+	-
Ruegeria faecimaris DSM 28009	+	-	+	-	+	+	-	-
Collimonas fungivorans Ter331	+	-	-	-	+	+	-	-
Burkholderia cepacia GG4	+	-	-	-	+	-	+	-
Burkholderia territorii MSMB1499	+	-	-	-	+	-	+	-
Aeromonas hydrophila NF1	-	+	-	-	+	-	-	-
Plesiomonas shigelloides GN7	-	+	-	-	+	-	-	-
Variovorax sp. GV051	+	+	-	-	+	-	-	-
Sinorhizobium medicae WSM419	+	-	+	-	+	+	-	-
Sinorhizobium meliloti 1021	+	-	+	-	+	+	-	-
Terasakiella pusilla DSM 6293	+	+	-	-	+	-	-	-
Vibrio alginolyticus NBRC 15630	-	+	-	-	+	-	-	-

Vibrio diabolicus CNCM I-1629	-	+	-	-	+	-	-	-
Vibrio cyclitrophicus 1F97	-	+	-	-	+	-	-	-
Arthrobacter sp. YC-RL1	+	-	-	-	-	-	-	+
Streptomyces albulus CCRC 11814	+	-	-	-	-	-	+	+
Acidobacteriaceae bacterium S15	-	+	-	-	-	-	-	+
Roseobacter sp. MED193	-	-	+	-	-	+	-	-
Falsirhodobacter sp. alg1	-	-	+	-	-	+	-	-
Rhodococcus rhodnii LMG 5362	-	-	+	-	-	-	-	+
Rhodococcus fascians A22b	-	-	-	-	-	-	-	+
Ruegeria pomeroyi DSS-3	-	-	+	-	-	+	-	-
Streptomyces sulphureus L180	-	-	-	-	-	-	-	+

Table S4 List of primers used in this paper.

Primer	Sequence	Plasmid	Used For
AepSTU ArmA_fwd	ATTCGAGCTCGGTACCCGGGCTCGAGG TCACAGTCGATC	pkmob*sacB-aepSTU*	Cloning region A of PPUBIRD1_3891-3895 (*aepSTU*)
AepSTU ArmA_rev	GGTCATGTTAGCGACCTTGTACTTGC CTTTTTC	pkmob*sacB-aepSTU*	Cloning region A of PPUBIRD1_3891-3895 (*aepSTU*)
AepSTU ArmB_fwd	ACAAGGTCGCGAAGGTCAAGGCCGAC GAAA	pkmob*sacB-aepSTU*	Cloning region B of PPUBIRD1_3891-3895 (*aepSTU*)
AepSTU ArmB_rev	TAAAACGACGGCCAGTGCCAACACTG TGCGATGTAGGAGC	pkmob*sacB-aepSTU*	Cloning region B of PPUBIRD1_3891-3895 (*aepSTU*)
PhnWm-ArmA_fwd	TACGAATTCGAGCTCGGTACCCGGGA GCTACCTCCAGGCGCCC	pkmob*sacB-phnW*	Cloning region A of PPUBIRD1_3442 (*phnW*)
PhnWm-ArmA_rev	ACCACACCGATACCCCAGGAGCCCCA GTCCA	pkmob*sacB-phnW*	Cloning region A of PPUBIRD1_3442 (*phnW*)
PhnWm-ArmB_fwd	TGGGGCTCCTGGGGTATCGGTGTGGT CGGGG	pkmob*sacB-phnW*	Cloning region B of PPUBIRD1_3442 (*phnW*)
PhnWm-ArmB_rev	CGTTGTAAAACGACGGCCAGTGCCAC CGATCTTCAGGCCGCCT	pkmob*sacB-phnW*	Cloning region B of PPUBIRD1_3442 (*phnW*)
PhnX_ArmA_fwd	TACGAATTCGAGCTCGGTACCCGGGC ACTGCACGAAGCACTGC	pkmob*sacB-phnX-gm*	Cloning region A of PPUBIRD1_3443 (*phnX*)
PhnX_ArmA_rev	CTCTAGAGTCGACATCTGGGTGGGAG CGAATG	pkmob*sacB-phnX-gm*	Cloning region A of PPUBIRD1_3443 (*phnX*)
PhnX_Gent_fwd	CTCCCACCCAGATGTCGACTCTAGAGG ATCCCCGG	pkmob*sacB-phnX-gm*	Cloning of *gm* cassette for Δ*phnX:gm* knockout
PhnX_Gent_rev	GCCGGCAAACAGTTTGGCCGCGGCGTT GTGA	pkmob*sacB-phnX-gm*	Cloning of *gm* cassette for Δ*phnX:gm* knockout
PhnX_ArmB_fwd	ACGCCGCGGCCAAACTGTTTGCCGGCT CCCG	pkmob*sacB-phnX-gm*	Cloning region B of PPUBIRD1_3443 (*phnX*)
PhnX_ArmB_rev	CGACGGCCAGTGCCAAGCTTGCATGGG GCCGGTTGGCCAATTTC	pkmob*sacB-phnX-gm*	Cloning region B of PPUBIRD1_3443 (*phnX*)

AepP_ArmA_fwd	ATTCGAGCTCGGTACCCGGGGAACAGCACCAGCAGGATG	pkmob*sacB-aepP*	Cloning region A of PPUBIRD1_3219 (*aepP*)
AepP_ArmA_rev	CCGTGACGCCCTTGTCGAGCATGAAACCTG	pkmob*sacB-aepP*	Cloning region A of PPUBIRD1_3219 (*aepP*)
AepP_ArmB_fwd	GCTCGACAAGGGCGTCACGGTGTTCATCG	pkmob*sacB-aepP*	Cloning region B of PPUBIRD1_3219 (*aepP*)
AepP_ArmB_rev	TAAAACGACGGCCAGTGCCACGGGCCTTTGACCAGCGG	pkmob*SacB-aepP*	Cloning region B of PPUBIRD1_3219 (*aepP*)
pBB:AepP_fwd	GCTGCAGGAATTCGATATCAGCTGCCGTGCACGGCCAC	pBB:*aepP*-km	Cloning of PPUBIRD1_3219 (*aepP*) for complementation
pBB:AepP_rev	TACCGGGCCCCCCCTCGAGGTTAAGTACGCAGTGGCTTGATTGCCATGC	pBB:*aepP*-km	Cloning of PPUBIRD1_3219 (*aepP*) for complementation
pBB:AepXVW^{BIRD}_fwd	GCTGCAGGAATTCGATATCACGGCGCCAGGCCAGGTTG	pBB:*aepXVW*^{BIRD}-km	Cloning of PPUBIRD1_4925-4927 (*aepXVW*) for complementation
pBB:AepXVW^{BIRD}_rev	TACCGGGCCCCCCCTCGAGGTTACTGCTGGGCCACCTTCTCC	pBB:*aepXVW*^{BIRD}-km	Cloning of PPUBIRD1_4925-4927 (*aepXVW*) for complementation
pBB:PhnWX_fwd	CTATAGGGCGAATTGGAGCTGAGCATGGCCTGCGCTTC	pBB:*phnWX*-km	Cloning of PPUBIRD1_3442-3443 (*phnWX*) for complementation
pBB:PhnWX_rev	CGAATTCCTGCAGCCCGGGGCGTTCTAGCTCAGGTTTGTCG	pBB:*phnWX*-km	Cloning of PPUBIRD1_3442-3443 (*phnWX*) for complementation
pBB:PhnW^{prom}_fwd	CTATAGGGCGAATTGGAGCTGAGCATGGCCTGCGCTTC	pBB:*phnW*^{prom}*X*-km	Cloning of PPUBIRD1_3443 (*phnX*) under the control of the *phnW* promoter for complementation
pBB:PhnW^{prom}_rev	TGTAGTTCATTCCGTTATTCCTCACAAGCGG	pBB:*phnW*^{prom}*X*-km	Cloning of PPUBIRD1_3443 (*phnX*) under the control of the *phnW* promoter for complementation
pBB:PhnX_fwd	GAATAACGGAATGAACTACAACAACCCC	pBB:*phnW*^{prom}*X*-km	Cloning of PPUBIRD1_3443 (*phnX*) under the control of the *phnW* promoter for complementation

pBB:PhnX_rev	CGAATTCCTGCAGCCCGGGGCGTTCTA GCTCAGGTTTG	pBB:*phnW*^{prom}*X*-km	Cloning of PPUBIRD1_3443 (*phnX*) under the control of the *phnW* promoter for complementation
pBB:AepX^{prom}_fwd	CCACCGCGGTGGCGGCCGCTGATTT CTGCTGGCTTCTTCG	pBB:*aepX*^{BIRDprom}*:* *aepXVW*^{Stappia}-km	Cloning of G572DRAFT_0252-0254 (*aepXVW Stappia stellulata*) under the control of the PPUBIRD1_4927 (*aepXVW*) promoter for complementation
pBB:AepX^{prom}_rev	CCGTTCCCATGGCATGGCCTCATC GAAG	pBB:*aepX*^{BIRDprom}*:* *aepXVW*^{Stappia}-km	Cloning of G572DRAFT_0252-0254 (*aepXVW Stappia stellulata*) under the control of the PPUBIRD1_4927 (*aepXVW*) promoter for complementation
pBB:AepXVW^{Stappia}_fwd	AGGCCATGCCATGGGAACGGTATCA AAGACTTTCGGAC	pBB:*aepX*^{BIRDprom}*:* *aepXVW*^{Stappia}-km	Cloning of G572DRAFT_0252-0254 (*aepXVW Stappia stellulata*) under the control of the PPUBIRD1_4927 (*aepXVW*) promoter for complementation
pBB:AepXVW^{Stappia}_rev	CGAATTCCTGCAGCCCGGGGTCAGC CCGGATCGCGCTG	pBB:*aepX*^{BIRDprom}*:* *aepXVW*^{Stappia}-km	Cloning of G572DRAFT_0252-0254 (*aepXVW Stappia stellulata*) under the control of the PPUBIRD1_4927 (*aepXVW*) promoter for complementation
pET21:AepXSs_Fwd	ATCTCAGTGGTGGTGGTGGTGGTG CTCGAG<u>CTTCGGCGCGGACTTCGA</u>	pET21a:*aepXSs*-amp	Cloning *S. stellulata aepX* into the pET21a expression vector
pET21:AepXSs_Rev	TTTAACTTTAAGAAGGAGATCTAC ATATG<u>CGCGAGACAATCACCGTC</u>	pET21a:*aepXSs*-amp	Cloning *S. stellulata aepX* into the pET21a expression vector
