# Supplementary material for: Transporter characterisation reveals aminoethylphosphonate mineralisation as a key step in the marine phosphorus redox cycle
Source: Nat Commun. 2021 Jul 27;12:4554. doi: 10.1038/s41467-021-24646-z (PMC8316502; doi:10.1038/s41467-021-24646-z)
Supplement: Supplementary file 4 — Description of additional supplementary files [file 41467_2021_24646_MOESM4_ESM.docx]

Description of additional supplementary information

Title: Supplementary Dataset 1

Description: Detailed proteomic datasets for P. putida and S. stellulata after analysis in MaxQuant and Perseus.
