# Supplementary material for: Transporter characterisation reveals aminoethylphosphonate mineralisation as a key step in the marine phosphorus redox cycle
Source: Nat Commun. 2021 Jul 27;12:4554. doi: 10.1038/s41467-021-24646-z (PMC8316502; doi:10.1038/s41467-021-24646-z)
Supplement: Supplementary file 5 — Reporting Summary [file 41467_2021_24646_MOESM5_ESM.pdf]

nature research

Corresponding author(s): Ian Lidbury

Last updated by author(s): Jun 18, 2021

Reporting Summary

Nature Research wishes to improve the reproducibility of the work that we publish. This form provides structure for consistency and transparency in reporting. For further information on Nature Research policies, see our Editorial Policies and the Editorial Policy Checklist.

Statistics

For all statistical analyses, confirm that the following items are present in the figure legend, table legend, main text, or Methods section.

n/a	Confirmed	
☐	☒	The exact sample size (*n*) for each experimental group/condition, given as a discrete number and unit of measurement
☐	☒	A statement on whether measurements were taken from distinct samples or whether the same sample was measured repeatedly
☐	☒	The statistical test(s) used AND whether they are one- or two-sided<br>*Only common tests should be described solely by name; describe more complex techniques in the Methods section.*
☒	☐	A description of all covariates tested
☐	☒	A description of any assumptions or corrections, such as tests of normality and adjustment for multiple comparisons
☐	☒	A full description of the statistical parameters including central tendency (e.g. means) or other basic estimates (e.g. regression coefficient) AND variation (e.g. standard deviation) or associated estimates of uncertainty (e.g. confidence intervals)
☐	☒	For null hypothesis testing, the test statistic (e.g. *F*, *t*, *r*) with confidence intervals, effect sizes, degrees of freedom and *P* value noted<br>*Give P values as exact values whenever suitable.*
☒	☐	For Bayesian analysis, information on the choice of priors and Markov chain Monte Carlo settings
☒	☐	For hierarchical and complex designs, identification of the appropriate level for tests and full reporting of outcomes
☒	☐	Estimates of effect sizes (e.g. Cohen's *d*, Pearson's *r*), indicating how they were calculated

Our web collection on statistics for biologists contains articles on many of the points above.

Software and code

Policy information about availability of computer code

Data collection	Genomic data was collected from the Integrated Microbial Genomes Database at the Joint Genome Institute (IMG/JGI).
Data analysis	All data analyses were performed using R (v 4.02) in the latest version of R Studio (v 1.3) using appropriate packages with standard code retrieved from various publicly available online sources. Alignments were performed using Clustal Omega (v 1.2.4). For phylogenetic analyses of proteins relationships were determined using IQTREE 1.6.12 and trees visualized using the Interactive Tree Of Life (ITOL) Online server (v 5.6.3). Proteomics data was analysed using Maxquant (v 1.6.17.0) and Perseus (v 1.6.12)

For manuscripts utilizing custom algorithms or software that are central to the research but not yet described in published literature, software must be made available to editors and reviewers. We strongly encourage code deposition in a community repository (e.g. GitHub). See the Nature Research guidelines for submitting code & software for further information.

Data

Policy information about availability of data

All manuscripts must include a data availability statement. This statement should provide the following information, where applicable:

- Accession codes, unique identifiers, or web links for publicly available datasets
- A list of figures that have associated raw data
- A description of any restrictions on data availability

The single-culture proteomic data generated in this study have been deposited in the ProteomeXchange Consortium via the PRoteomics IDEntifications (PRIDE) database with the dataset identifier PXD026804 [doi:10.6019/PXD026804]. The TARA Oceans metagenomic and metatranscriptomic data used in this study are available in the European Nucleotide Archive database under accession code PRJEB7988 [https://www.ebi.ac.uk/ena/browser/view/PRJEB7988].

Field-specific reporting

Please select the one below that is the best fit for your research. If you are not sure, read the appropriate sections before making your selection.

☒ Life sciences ☐ Behavioural & social sciences ☐ Ecological, evolutionary & environmental sciences

For a reference copy of the document with all sections, see nature.com/documents/nr-reporting-summary-flat.pdf

Life sciences study design

All studies must disclose on these points even when the disclosure is negative.

Sample size	Microbial growth experiments, proteomics experiments, and substrate binding affinity assays were performed with samples sizes of 3-4 replicates, as is standard in microbiology. No further sample size calculation was performed. Environmental data analysis was performed using all publicly available samples linked to the TARA Oceans database, as such no sample size calculation was performed. This dataset consists of 180 and 190 metagenomic and metatranscriptomic sampling sites, respectively. All metagenomic and all metatranscriptomic sites are biologically independent within their respective categories, and as such the dataset is sufficiently robust for the questions asked of it.
Data exclusions	No data was excluded.
Replication	Replication for laboratory cultures were performed in triplicate or quadruplicate as per the current standard of practice for defined microbial growth assays. Transporter knockout mutants were repeatedly used in multiple experiments, demonstrating reproducibility of their phenotypes. Additionally, complementation experiments were performed to confirm the wild-type phenotype could be restored with the presence of the knocked out gene alone. These growth experiments were thus successfully replicated. Proteomic analyses were also performed in triplicate samples form three independent culture replicates, however these experiments were not performed more than once, for reasons of cost.
Randomization	Randomization is not applicable to microbial growth experiments which involve clonal populations and tight control of all biotic and abiotic factors other than those under investigation. As such, randomization, the purpose of which is to control for genetic and environmental variability, is uneccessary. Consequently, randomization is not part of the current standard of practice for defined microbial growth assays. Since the proteomics experiments were performed on microbial growth cultures, the same rationale applies. Sampling sites within the pre-existing TARA ocean dataset were designed with the aim of sampling from a wide variety of oceanic environments, including coastal shelf and oceanic gyre regions. As sampling required transport by ship, randomizing site locations would be impractical.
Blinding	Deidentification of cultures is not part of the current standard of practice for defined microbial growth assays. Most growth experiments were performed in 96-well plate readers which collect data automatically, and so data was only examined by researchers at the point of analysis. The pre-existing TARA ocean dataset could not be blinded given it was complete at the point of analysis.

Reporting for specific materials, systems and methods

We require information from authors about some types of materials, experimental systems and methods used in many studies. Here, indicate whether each material, system or method listed is relevant to your study. If you are not sure if a list item applies to your research, read the appropriate section before selecting a response.

Materials & experimental systems

n/a	Involved in the study	
☒	☐	Antibodies
☒	☐	Eukaryotic cell lines
☒	☐	Palaeontology and archaeology
☒	☐	Animals and other organisms
☒	☐	Human research participants
☒	☐	Clinical data
☒	☐	Dual use research of concern

Methods

n/a	Involved in the study	
☒	☐	ChIP-seq
☒	☐	Flow cytometry
☒	☐	MRI-based neuroimaging
